# Nursing care for users with post-COVID conditions in primary and specialized health care

**DOI:** 10.1590/1980-220X-REEUSP-2025-0043en

**Published:** 2025-12-12

**Authors:** Rafaela Deharo Curvelo, Ana Cristina Ribeiro La Scaléa, Sílvia Carla da Silva André Uehara

**Affiliations:** 1 Universidade Federal de São Carlos, Centro de Ciências Biológicas e da Saúde, Departamento de Enfermagem, São Carlos, SP, Brazil.

**Keywords:** Nurse Practitioners, Post-Acute COVID-19 Syndrome, Primary Health Care, Secondary Care

## Abstract

**Objective:**

To analyze and compare the nursing care provided to users with Post-COVID Conditions (PCC) in Primary Health Care (PHC) and Specialized Care (SC).

**Method:**

This is an analytical cross-sectional study carried out with 226 nursing professionals from PHC and SC in Brazil. Data were collected through a self-responding form published by the Nursing Councils and on social media and were analyzed using descriptive statistics, followed by the t-test to compare means and chi-square tests to study associations with categorical data. The study was approved by the UFSCar Research Ethics Committee.

**Results:**

An association between PHC nursing professionals and the identification of persistent cough as the most common symptom of PCC (80.2% vs 50%; p< 0.01) and neurocognitive deficit as the most severe symptom (62% vs 42.1%; p = 0.04); and an association between SC professionals and the identification of loss of appetite as the least common symptom of PCC (43.7% vs 22%; p = 0.02).

**Conclusion:**

The results highlight challenges such as the difficulty in identifying PCC, the absence of widely used protocols, and the limited training of PHC and SC nursing professionals, impacting the quality of care.

## INTRODUCTION

The Post-COVID Condition or long COVID was defined by the World Health Organization (WHO) as the presence of persistent signs and symptoms that affect individuals after acute COVID-19 infection among 10% to 20% of people recovered from the disease^(1)^. This study considers the concept of “post-COVID condition” (PCC), defined in technical note no. 57/2023 of the Ministry of Health as the presence of “signs, symptoms and/or conditions that continue or develop four weeks or more after the initial infection by SARS-CoV-2 and cannot be justified by an alternative diagnosis”^(2)^.

In this context, PCC is a chronic systemic inflammation that can present symptoms such as fatigue, dyspnea, neurological and psychiatric impairments, cough, muscle and joint pain, changes in smell and taste, in addition to other multifactorial clinical manifestations, which can improve, worsen, or reappear over time. Therefore, as it is a complex and disabling condition that affects daily activities due to the diversity of symptoms, with an unclear etiology, sensitivity is required to perform the appropriate diagnosis and management, increasing the challenges for health services^(2)^.

To achieve this, an integrated and multidisciplinary approach is essential, with a complete and individualized care plan. Thus, Primary Health Care (PHC), considered the main gateway to the Brazilian Public Health System (*SUS*), carries out the initial assessment and management of PCC cases to ensure continuous and comprehensive care. Furthermore, more complex cases should be referred to Specialized Care (SC), optimizing the resources of the Health Care Network (*RAS*) and addressing chronic and serious conditions efficiently^(3)^.

As a result of such heterogeneity, nursing, as it comprises the class of professionals in constant contact with health service users, has an essential role in embracing and caring for people with PCC, since it must integrate health education actions, direct assistance, and coordination with social support networks^(4)^. It is recommended, as a PCC management strategy, physical rehabilitation as prophylaxis and direct treatment, monitoring of pre-existing comorbidities to avoid worsening and risk factors for the development of the condition, providing mental health support and, for those facing social and financial difficulties, providing support from social services^(5, 6)^.

Considering that PHC is responsible for initial and ongoing care, while SC works on managing more complex cases, it is essential to understand how nursing care is organized at each level of care, with a focus on comprehensive care. Moreover, given the scarcity of studies that specifically address the role of nursing in the context of PCC at different points in the network, the comparative analysis between these levels allows identifying care gaps, strengthening evidence-based practices, and promoting more efficient use of resources available in the RAS. Additionally, it is worth highlighting the lack of studies that address nursing care directed at people with PCC at any level of care.

Thus, the present study aimed to analyze and compare the nursing care provided to users with PCC in PHC and SC.

## METHOD

### Design of Study

This is an analytical cross-sectional study, guided by the recommendations of the Strengthening the Reporting of Observational Studies in Epidemiology (STROBE)^(7)^.

### Population, Local and Selection Criteria

For this study, a convenience sample of 226 nursing professionals was used, 163 PHC workers and 63 SC workers, distributed throughout Brazil. Considering the increase in the number of professionals temporarily hired during the pandemic, especially during the most critical period, there was a direct impact on the definition of the population eligible for the study, justifying the use of a convenience sample, since the application of a sample calculation was not feasible.

The inclusion criteria were nurses, nursing technicians, and nursing assistants who worked at the institution during the COVID-19 pandemic for at least three months, and participants who were on leave and/or vacation during this period were excluded.

### Data Collection

The data collection instrument was initially prepared in a Word document, based on the Manual for Assessment and Management of Post-COVID Conditions in Primary Health Care, from the Ministry of Health^(8)^. The questions in the self-administered questionnaire were developed by the researchers based on a direct adaptation of the manual’s guidelines and instructions, focusing on practical applicability for the study’s target audience. The questions addressed aspects such as the definition of PCC, clinical manifestations, return to activities, additional tests, assessment, clinical management, and referral to specialized services. The questionnaire, consisting of 20 closed-ended questions with yes/no or multiple-choice answers, was then made available to participants through the Google Forms platform, which ensured practicality and facilitated remote access to data collection.

Data collection was carried out from May 25, 2023 to January 22, 2024, and the research was widely publicized by Regional Nursing Councils and on social media. This measure was adopted to try to reach as many professionals as possible from all Brazilian regions, ensuring the same chance of response to all of them.

### Data Analysis and Treatment

After data collection was completed, the responses were tabulated in Excel, organized in chronological order (from the beginning of data collection) and used for statistical analysis purposes. Initially, a descriptive analysis of the data was carried out, using absolute and percentage frequencies (qualitative variables) in general and by type of unit (PHC and SC).

Furthermore, data were transported to the software SAS 9.4, where the data analysis was performed^(9)^. Thus, the associations between the type of unit and the variables of interest were analyzed using Fisher’s exact test^(10)^. For all analyses, a significance level of 5% was adopted.

Unanswered responses were included in the final analysis, but were considered, in the data analysis, as absent and identified within each question on the form.

### Ethical Aspects

The study was approved by the Research Ethics Committee (*CEP*) of the *Universidade Federal de São Carlos* (UFSCar), with Certificate of Presentation of Ethical Appreciation (CAAE) no. 68157323.6.0000.5504 and Opinion Number 6, 244, 667. Data collection began after the participant signed the Free and Informed Consent Form (FICF).

## RESULTS

A total of 226 nursing professionals participated in the study, 91.1% (205) of whom were nurses. Of these, 52.4% (98) declared themselves to be white, 46.5% (87) declared themselves to be black, and 1.1% (2) declared themselves to be yellow, with 39 not responding to the questions.

The results showed that no statistical differences were identified regarding the care provided by nursing professionals from PHC and SC for PCC (Table 1).

**Table 1:** Knowledge and conceptualization of PCC by nursing professionals – São Carlos, SP, Brazil, 2024.

Variable	Specialized care (n = 63)	Primary care (n = 163)	p-value^**^
Have you heard of post-COVID condition?			0, 87
No	19 (30.6%)	48 (29.4%)	
Yes	43 (69.3%)	115 (71.5%)	
Did not answer = 1	1	0	
Do you know how to conceptualize post-COVID conditions?			0.65
No	30 (48.4%)	72 (44.2%)	
Yes	32 (51.6%)	91 (55.8%)	
Did not answer = 1	1	0	

**Fisher’s exact test.

Source: Prepared by the author, 2023.

When performing the comparative analysis between professionals from different services, an association stands out between PHC nursing professionals and the reference to coughing as a common symptom of PCC and to neurocognitive deficit as a serious symptom. An association was also observed between being a SC professional and the identification of lack of appetite among the less common clinical manifestations of the post-COVID condition (Table 2).

**Table 2:** Clinical manifestations of post-COVID conditions cited by nursing professionals – São Carlos, SP, Brazil, 2024.

Variable	Specialized care (n = 63)	Primary care (n = 163)	p-value^**^
Do you know what the most common clinical manifestations of post-COVID conditions are?			0.27
No	17 (27%)	57 (35%)	
Yes	46 (73%)	106 (65%)	
Fatigue	38 (82.6%)	91 (85.8%)	0.63
Cough	23 (50%)	85 (80.2%)	< 0.01
Dyspnea	26 (56.5%)	76 (71.7%)	0.09
Anosmia (changes in smell)	28 (60.9%)	69 (65.1%)	0.71
Chest discomfort	22 (47.8%)	66 (62.3%)	0.11
Do you know what the less common clinical manifestations of post-COVID conditions are?^*^			0.55
No	31 (49.2%)	72 (44.2%)	
Yes	32 (50, 8%)	91 (55.8%)	
Alopecia	16 (50%)	45 (49.4%)	0.99
Sweating	14 (43.7%)	45 (49.4%)	0, 68
Insomnia	13 (40.6%)	35 (38.5%)	0, 84
Diarrhea	8 (25%)	37 (40.7%)	0.14
Arthralgia and/or myalgia	13 (40.6%)	28 (30.8%)	0, 38
Vertigo	10 (31.2%)	31 (34.1%)	0.83
Dizziness	7 (21.9%)	28 (30.8%)	0, 37
Loss of appetite	14 (43.7%)	20 (22%)	0.02
Headache	9 (28.1%)	24 (26.4%)	0.82
Rhinorrhea	7 (21.9%)	25 (27.5%)	0.64
Dysgeusia (taste disorders)	6 (18.7%)	16 (17.6%)	0.99
Do you know what the neuropsychiatric clinical manifestations of the post-COVID condition are?			0.65
No	21 (33.3%)	60 (36.8%)	
Yes	42 (66.7%)	103 (63.2%)	
Memory loss	30 (71.4%)	82 (80.4%)	0.27
Reduction of concentration	29 (69%)	75 (72.8%)	0.69
Anxiety/depression	27 (64.3%)	73 (71.6%)	0.43
Post-traumatic stress disorder	15 (35.7%)	45 (44.1%)	0.46
Did not answer = 1	0	1	
Do you know what the most serious clinical manifestations of the post-COVID condition are?			0.30
No	24 (38.1%)	75 (46%)	
Yes	39 (61.9%)	88 (54%)	
Pulmonary fibrosis	26 (68.4%)	60 (68.2%)	0.99
Thromboembolic disorders	25 (65.8%)	55 (62%)	0.84
Neurocognitive deficit	16 (42.1%)	55 (62%)	0.04
Sequelae of viral myocardial damage with reduced systolic function and arrhythmias	19 (50%)	41 (46.6%)	0.85
Guillain-Barré syndrome	10 (26.3%)	23 (26.1%)	0.99
Rash with different possible presentations (vesicular, maculopapular, urticarial, or similar to erythema pernio)	6 (15.8%)	18 (20.4%)	0.63
Did not answer = 1	1	0	

**Fisher’s exact test.

Source: Prepared by the author, 2023.

In addition, no statistical relationship was found between what was reported by PHC and SC professionals regarding active infection and diagnosis of post-COVID condition (Table 3).

**Table 3:** Nursing care provided by SC and PHC regarding active infection and PCC diagnosis – São Carlos, SP, Brazil, 2024.

Variable	Specialized care (n = 63)	Primary care (n = 163)	p-value^**^
Do you think it is possible to have an active viral infection in patients with PCC?			0.69
No	11 (17.7%)	25 (15.6%)	
Yes	51 (82.3%)	135 (84.4%)	
Did not answer = 4	1	3	
Do you think an individual with PCC is PCR positive?			0.99
No	24 (38.7%)	63 (39.6%)	
Yes	38 (61.3%)	96 (60.4%)	
Did not answer = 5	1	4	
Do you know how PCC is diagnosed?			0.76
No	36 (59%)	90 (55.9%)	
Yes	25 (41%)	71 (44.1%)	
Did not answer = 4	2	2	
Is it indicated to retest patients with PCC with tests to assess active SARS-CoV-2 infection (RT-PCR/RT-LAMP or antigen test) within the first 90 days after primary infection?			0.76
No	25 (41.7%)	60 (38.5%)	
Yes	35 (58.3%)	96 (61.5%)	
Did not answer = 10	3	7	
Do you know if cases that appear with new symptoms in the first 90 days of the primary infection, without another defined etiology, and, especially, if there was contact with a confirmed case, can be retested based on medical evaluation and epidemiological surveillance?			0.99
No	23 (37.7%)	58 (36.7%)	
Yes	38 (62.3%)	100 (63.3%)	
Did not answer = 7	2	5	
If the individual presents new symptoms compatible with flu-like syndrome, after 90 days, is new testing with viral detection tests recommended?			0.42
No	8 (12.9%)	29 (18.5%)	
Yes	54 (87.1%)	128 (81.5%)	
Did not answer = 7	1	6	

**Fisher’s exact test.

Source: Prepared by the author, 2023.

Statistical analyses found no association between the research participant’s unit of origin and the indications for additional tests and assistance actions for users with post-COVID conditions (Table 4).

**Table 4:** Nursing care in SC and PHC for people with PCC – São Carlos, SP, Brazil, 2024.

Variable	Specialized care (n = 63)	Primary care (n = 163)	p-value^**^
Is it recommended to perform additional tests in all cases of patients with PCC?			0.45
No	23 (36.5%)	69 (42.3%)	
Yes	40 (63.5%)	94 (57.7%)	
What are the clinical situations that require additional tests for the evaluation of PCC?^*^			
Patients recovering from serious illness, discharged after hospitalization, previously identified laboratory abnormalities, or those with persistent unexplained symptoms.	34 (54%)	106 (65%)	0.13
Disease complicated by heart failure or myocarditis, or with their signs and symptoms, such as dyspnea, chest discomfort, edema.	34 (54%)	99 (60.7%)	0, 37
Unexplained fatigue or weakness	29 (46%)	69 (42.3%)	0.65
Arthralgia, myalgia and other signs/symptoms compatible with rheumatological disease.	23 (36.1%)	55 (33.7%)	0.76
Recrudescence of fever.	12 (19%)	46 (28.2%)	0.18
What care actions are necessary for a patient with PCC?^*^			
Assessment and management of comorbidities	52 (82.5%)	129 (79.1%)	0.71
Attention to general health care: diet, avoiding smoking and alcohol, quality of sleep.	27 (42.9%)	91 (55.8%)	0.10
Mental health care	27 (42.9%)	82 (50.3%)	0.37
Gradual increase in physical exercise	16 (25.4%)	62 (38%)	0.09

*It allows multiple answers, so the sum of the percentages will not be 100%

**Fisher’s exact test.

Source: Prepared by the author, 2023.

An association was also found between the origin of the healthcare professional and what was reported about the need to refer PCC cases with persistent dyspnea or cough after 12 weeks of the acute onset of COVID-19 (Table 5).

**Table 5:** Comparison between the health professionals’ service of origin and the conditions for referrals of people with PCC – São Carlos, SP, Brazil, 2024.

Variable	Specialized care (n = 63)	Primary care (n = 163)	p-value^**^
Under what conditions do patients with PCC need to be referred to specialized services?^*^			
Suspected or diagnosed complications such as thromboembolism, acute myocardial infarction, pericarditis, myocarditis, decompensated heart failure, or acute neurological event	37 (58.7%)	106 (65%)	0.44
Patients who have had severe lung impairment, pulmonary fibrosis, or prolonged mechanical ventilation	33 (52.4%)	97 (59.5%)	0.37
Patient with a history of post-COVID pulmonary thromboembolism who continues to experience dyspnea, fatigue, exercise intolerance, dizziness, and/or syncope 12 weeks after the thromboembolic event	34 (54%)	87 (53.4%)	0.99
Persistent respiratory symptoms after 12 weeks	30 (47.6%)	78 (47.8%)	0.99
Persistent dyspnea or cough unresponsive to optimized clinical treatment after 12 weeks of acute COVID-19	19 (30.2%)	77 (47.2%)	0.02
Acute coronary syndrome in the context of COVID-19 infection	24 (38.1%)	70 (42.9%)	0,55
Depression, anxiety and/or post-traumatic stress disorder	27 (42.9%)	63 (38.6%)	0.65
Suspected myocarditis	25 (39.7%)	63 (38.6%)	0.88
New diagnosis of heart failure or symptomatic worsening of preexisting heart failure	22 (34.9%)	63 (38.6%)	0.65
Patients with mild to moderate COVID-19 who continue to have dyspnea 12 weeks after the initial onset	22 (34.9%)	55 (33.7%)	0.88
Risk of suicide or heteroaggression, risk of moral exposure and exacerbated psychotic symptoms	24 (38.1%)	51 (31.3%)	0.35
Need to maintain oxygen therapy after 4 weeks of hospital discharge	16 (25.4%)	59 (36.2%)	0.16
Pericarditis, after evaluation in the emergency room, if symptoms persist after adequate treatment with nonsteroidal antiinflammatory drugs	19 (30.2%)	53 (32.5%)	0.87
Cognitive decline and/or polyneuropathy	16 (25.4%)	54 (33.1%)	0.34
Recurrent palpitation of undetermined origin	13 (20.6%)	53 (32.5%)	0.10
Symptomatic or recurrent supraventricular tachycardia	15 (23.8%)	51 (31.3%)	0.33
Atrial fibrillation with possibility of cardioversion	15 (23.8%)	30 (28.8)	0.51
Suspected postural orthostatic tachycardia syndrome	11 (17.5%)	46 (28.2%)	0.12
Atrial flutter	9 (14.3%)	41 (25.1%)	0.11

*It allows multiple answers, so the sum of the percentages will not be 100%

**Fisher’s exact test.

Source: Prepared by the author, 2023.

## DISCUSSION

The results indicate a gap in the care provided by nurses in the SC and PHC sectors regarding PCC, which may be related to the fact that it is a new disease or to the complexity of identification, as well as the lack of training provided by the services and the absence of effective implementation of guidelines aimed at caring for the disease.

In this scenario, the identification of PCC in health services is hampered by its systemic nature and the variability of symptoms, which often differ from those observed in the acute phase of SARS-CoV-2 infection^(11, 12)^. The most common symptoms of PCC are fatigue, exercise intolerance, dyspnea, and myalgia, while less frequent manifestations include diarrhea, abdominal pain, anorexia, changes in smell and taste, sore throat, weight gain or loss, cough, palpitations, and chest discomfort^(13)^.

The persistence of these symptoms may be related to acute respiratory symptoms and prolonged hospitalization during the acute phase of COVID-19. Severe forms of COVID-19, which often result in prolonged hospitalizations, can trigger intense immune responses and organ damage. Additionally, aggressive treatments, intubation, and nosocomial infections associated with the management of acute respiratory symptoms may also contribute to persistent signs and symptoms^(13)^. Furthermore, the similarity with other post-infectious conditions, such as myalgic encephalomyelitis/chronic fatigue syndrome and fibromyalgia, influence the definition of the diagnosis of PCC^(14)^.

It is important to highlight that the identification of PCC symptoms can vary significantly between different levels of health care. PHC professionals maintain continuous and direct contact with patients within their coverage area, whether through regular consultations or home visits. This proximity to the user favors the early and recurrent identification of more common symptoms, such as persistent cough, which are frequently reported by patients and easily observed by nursing professionals, especially in post-infection surveillance contexts^(15, 16)^.

However, neurocognitive deficits, such as difficulty concentrating, brain fog, and memory loss, can be interpreted as more serious manifestations due to their direct impact on functionality and quality of life, interfering with daily life. Added to this is the growing dissemination in the literature of neurological sequelae associated with COVID-19 infection, which may contribute to expanding the understanding of the severity of symptoms^(15, 16)^.

Conversely, SC is more focused on specific care, predominantly directed at cases of greater clinical complexity, with a focus on specific conditions that require more targeted diagnostic and therapeutic interventions. In this context, nonspecific symptoms tend to be undervalued or not prioritized during the consultation, compared to more evident or disabling manifestations^(17)^. Thus, differences in clinical approach and nurses’ bond within PHC and SC influence the identification of the severity of PCC symptoms, with PHC favoring the observation of subtle changes and SC focusing on more complex cases^(18, 19)^.

In the context of PHC, especially, nurses play a multidimensional role in providing assistance and managing care in direct contact with users. However, these professionals face significant challenges in implementing guidelines, facilitating access to health services, and providing targeted support^(20)^. Furthermore, when faced with clinical cases of PCC that cannot be resolved in PHC and need to be referred to specialized levels of health care, they also face difficulties associated with clinical uncertainty, which results in fragmented and poorly coordinated care provision and a lack of multidisciplinary approaches, neglecting the psychosocial needs of users and focusing on the biomedical model^(21)^.

In this context, the lack of integration between levels of care, combined with the absence of clear referral and counter-referral flows, contributes to delayed diagnoses and the overload of the SC. In this regard, the adoption of strategies such as the use of electronic medical records, the standardization of clinical criteria for risk stratification, and the creation of specialized outpatient clinics to facilitate early identification and appropriate referral of cases is proposed. Furthermore, the return of clinical information to the PHC through counter-referral is equally important to ensure the continuity and resolution of care^(22)^.

In the Brazilian scenario, there is notable difficulty in regulation between PHC and SC, with a long waiting list for consultations and exams caused by the limited resolution of demands in PHC. Furthermore, underreporting of symptoms in medical records has been a recurring issue, which may occur due to a lack of adequate communication by users or inadequate description by healthcare professionals. Thus, the need for integrated and multidisciplinary approaches in PHC is highlighted, especially to deal with complex PCC^(16)^.

In a study conducted in Belgium, PHC professionals reported that easy access to materials from the scientific literature or management protocols for PCC is insufficient and limited, with the duration of signs and symptoms being the most common diagnostic criterion for identifying cases. Moreover, insufficient knowledge of the care and identification of PCC cases in the service is related to the lack of training or information on the part of the work or teaching institution about the disease^(23)^.

In Brazil, the existence of the Manual for the assessment and management of post-COVID conditions in Primary Health Care, developed by the Ministry of Health, stands out; however, there is a lack of dissemination of this material in services, in view of the difficulties of nursing professionals in identifying the common clinical manifestations of the disease, management, and criteria for referral to specialized services^(8)^. In this context, publishing material does not guarantee that it will reach professionals or that they will use it appropriately, since the absence of continuing education strategy programs hinders access and influences the appropriate application of these guidelines^(20)^.

Therefore, it is important to highlight that the lack of knowledge about the clinical manifestations and complexity of PCC among nursing professionals compromises the ability to perform early detection, adequate monitoring, and appropriate clinical management of affected patients. Given the multifactorial and often nonspecific nature of symptoms, the lack of specific knowledge can lead to underappreciation of complaints, delays in diagnosis, and failures in the management of cases that require ongoing care. This gap negatively impacts the quality of care, especially in PHC, where nursing acts as the initial point of contact and a central element in coordinating care^(17,18,19)^.

Therefore, the need for investment in continuing nursing training and the incorporation of the Ministry of Health’s manual on PCC into the care routine in PHC is emphasized, which can be operationalized through the use of standardized clinical checklists. This way, the systematic use of this manual, combined with the integration of PHC and SC, constitutes a structuring strategy to qualify care for people with PCC in the Brazilian health system^(22)^.

Consequently, the challenge of nursing care is not limited to the nurse-user relationship, but also involves the care and adaptation of the professionals themselves. Additionally, interprofessional collaboration and the active involvement of users in their care are also essential, but there is a considerable lack of specialists in the area, in addition to the limitation of continuing education activities planned by managers, especially at the municipal level^(20, 24)^.

In general, the work of the nursing team at all levels of care is considered fundamental to providing comprehensive care to users with PCC, including actions for users’ identification and monitoring, guidance and support, performance of health education activities, participation in clinical research and coordination of care in a multidisciplinary manner, together with general practitioners, specialist doctors, and physiotherapists^(24)^.

In the context of PHC, the health needs identified by the nursing team must be met in a planned manner, including home consultations. Furthermore, professionals should support caregivers in managing PCC and other underlying comorbidities. Likewise, encouraging the creation of health education groups for users with PCC contributes to self-care and knowledge of the prolonged symptoms of COVID-19^(25)^.

In contrast, SC, which covers medium-complexity technology services, must have a multidisciplinary team (psychologist, nurse, physiotherapist, nutritionist and occupational therapist) to provide examinations, diagnostic tests, and therapeutic strategies for managing PCC symptoms. Thus, the competencies of SC include welcoming the user, understanding their needs, and carrying out comprehensive multidisciplinary assessments, ideally through a Singular Therapeutic Project (*PTS*) to identify individualized care. Also, care for those affected by PCC must be shared with the PHC, considering the clinical evolution and the needs of users, and severe cases must be referred to emergency services^(26, 27)^.

Therefore, referral must be carried out considering biopsychosocial factors, aiming to contemplate multidisciplinarity, and the nursing professional, in addition to recognizing any referral, has to develop a personalized rehabilitation and health management plan for each individual, considering the family and the community^(25, 28)^.

In this sense, there is a need to develop care protocols for health services (especially SC, since there is little scientific production on the management of post-COVID conditions at this level of health care), aiming to facilitate communication between professionals and levels of care, in addition to ensuring quality in decision-making, preventing risks, and promoting innovations in nursing care^(29)^.

This study highlights the need to develop strategies for the implementation of existing and new protocols, which aim to organize the clinical management and referral criteria for PCC, given the scarcity in the literature. Furthermore, the need for the nursing team to recognize itself as coordinator of care for those affected by PCC and creator of individualized care plans, with an emphasis on the biopsychosocial management of the user, as well as the urgent need to strengthen continuing education policies, especially at the municipal level, is highlighted.

Finally, a limitation of this study is the low participation of nursing professionals in the research, even though it was carried out nationally and was widely publicized, especially by the professional council. In addition, the study explored nurses’ knowledge through self-reports, which may not accurately reflect actual technical knowledge, as it is subject to recall bias, social desirability, or overestimation of respondents’ knowledge and clinical practice. Furthermore, the use of convenience sampling may have restricted the representativeness of participants, limiting the generalizability of the results. Despite this, the study presents robust results, contributing significantly to the understanding of the challenges faced by nursing professionals in identifying and managing PCC, reinforcing the need for structured training and care organization strategies.

## CONCLUSION

The results of this study indicate specific differences in what was reported by PHC and SC nurses, especially regarding the identification of specific clinical manifestations and the need for referral in cases of persistent symptoms. However, no significant difference was found in the responses obtained between professionals at different levels of care in relation to diagnosis, additional tests or assistance actions.

In addition, when analyzing the results, a “basic” knowledge about the disease (i.e., common and easily identified concepts and manifestations) and its management, diagnosis and identification is observed, since the responses cover the care and recognition of COVID-19, but not necessarily of PCC. Furthermore, there is a lack of knowledge about PCC, as many nursing professionals face difficulties in recognizing the symptoms, which can be confused with other conditions.

Thus, the identification and clinical management of PCC are hampered by the absence of widely publicized protocols, the lack and insufficient training of nursing professionals, and the fragile integration between levels of health care. These factors compromise the quality of care provided, both in PHC and SC, favoring fragmented approaches and hindering an effective response to users’ needs.

The findings of this study highlight the central role of nursing in coordinating care for people with PCC, highlighting the urgency of developing continuing education and continuous training strategies for the nursing team, with a specific focus on PCC. This can ensure the effective implementation of referral flows, promoting more integrated and effective care.

Furthermore, integrated and multidisciplinary actions, as well as the strengthening of PHC as a gateway to health services, are fundamental ways to improve the excellence of care and meet the users’ needs. Therefore, this study contributes to future research and the development of public policies that seek to guarantee quality, integrated, and accessible care for those affected by PCC in Brazil.

## DATA AVAILABILITY

All the data that supports the results from this study were published in this article.
