# Supplementary material for: Nursing care for users with post-COVID conditions in primary and specialized health care
Source: Rev Esc Enferm USP. 2025 Dec 12;59:e20250043. doi: 10.1590/1980-220X-REEUSP-2025-0043en (PMC12704597; doi:10.1590/1980-220X-REEUSP-2025-0043en)
Supplement: Translation

# Translation: Cuidado de enfermagem aos usuários com condição pós-COVID na atenção primária e especializada à saúde

**DOI:** 10.1590/1980-220X-REEUSP-2025-0043pt

**Authors:** Rafaela Deharo Curvelo, Ana Cristina Ribeiro La Scaléa, Sílvia Carla da Silva André Uehara

**Affiliations:** 1 Universidade Federal de São Carlos, Centro de Ciências Biológicas e da Saúde, Departamento de Enfermagem, São Carlos, SP, Brasil.

**Keywords:** Profissionais de Enfermagem, Síndrome Pós-COVID-19 Aguda, Atenção Primária à Saúde, Atenção Secundária à Saúde

## Abstract

**Objetivo:**

Analisar e comparar o cuidado de enfermagem oferecido aos usuários com Condição Pós-Covid (CPC) da Atenção Primária à Saúde (APS) e da Atenção Especializada (AE).

**Método:**

Trata-se de um estudo transversal analítico realizado com 226 profissionais de enfermagem da APS e AE do Brasil. Os dados foram coletados por meio de um formulário autorrespondido divulgado pelos Conselhos de Enfermagem e nas redes sociais e foram analisados por meio da estatística descritiva, seguida pelo teste t para comparação de médias e testes de qui-quadrado para o estudo de associação com dados categóricos. O estudo foi aprovado pelo Comitê de Ética em Pesquisa da UFSCar.

**Resultados:**

Destacam-se uma associação entre profissionais de enfermagem da APS e a identificação de tosse persistente como sendo o sintoma mais comum da CPC (80, 2% vs 50%; p < 0, 01) e de déficit neurocognitivo como sintoma mais grave (62% vs 42, 1%; p = 0, 04); e uma associação entre profissionais da AE e a identificação de inapetência como o sintoma menos comum da CPC (43, 7% vs 22%; p = 0, 02).

**Conclusão:**

Os resultados evidenciam desafios como a dificuldade na identificação da CPC, ausência de protocolos amplamente utilizados e a capacitação limitada dos profissionais de enfermagem de APS e AE, impactando a qualidade do cuidado.

## INTRODUÇÃO

A Condição Pós-Covid ou Covid longa foi definida pela Organização Mundial da Saúde (OMS) como sendo a presença de sinais e sintomas persistentes que afetam indivíduos após a infecção aguda da Covid-19 entre 10% a 20% de pessoas recuperadas da doença^(1)^. Neste estudo, está sendo considerado o conceito de “condição pós-Covid” (CPC), definida na nota técnica nº57/2023 do Ministério da Saúde como a presença de “sinais, sintomas e/ou condições que continuam ou se desenvolvem quatro semanas ou mais após a infecção inicial pelo SARS-CoV-2 e não podem ser justificadas por um diagnóstico alternativo”^(2)^.

Nesse contexto, a CPC é uma inflamação crônica sistêmica que pode apresentar sintomas como fadiga, dispneia, acometimentos neurológicos e psiquiátricos, tosse, dores musculares e articulares, alterações no olfato e paladar, além de outras manifestações clínicas multifatoriais, que podem melhorar, agravar ou reaparecer com o tempo. Assim, por ser uma condição complexa e incapacitante que afeta as atividades diárias devido à diversidade de sintomas, com uma etiologia pouco clara, exige-se sensibilidade para a realização do diagnóstico e manejo adequados, potencializando os desafios para os serviços de saúde^(2)^.

Para isso, é essencial uma abordagem integrada e multidisciplinar, com um plano de cuidados completo e individualizado. Assim, a Atenção Primária à Saúde (APS), considerada a principal porta de entrada do Sistema Único de Saúde (SUS), realiza a avaliação e manejo iniciais dos casos de CPC de forma a garantir um cuidado contínuo e abrangente. Ademais, casos de maior complexidade devem ser encaminhados para a Atenção Especializada (AE), otimizando os recursos da Rede de Atenção à Saúde (RAS) e abordando acometimentos crônicos e graves de forma eficiente^(3)^.

Em decorrência de tal heterogeneidade, a enfermagem, por compreender a classe de profissionais em constante contato com os usuários dos serviços de saúde, tem um papel essencial no acolhimento e cuidado às pessoas com CPC, uma vez que deve integrar ações de educação em saúde, assistência direta e coordenação com redes de apoio social^(4)^. Recomenda-se, como estratégia de manejo de CPC, reabilitação física como profilaxia e tratamento direto, monitoramento das comorbidades pré-existentes para evitar agravamentos e fatores de risco para o desenvolvimento da condição, oferecer suporte à saúde mental e, entre aqueles que enfrentam dificuldades sociais e financeiras, ofertar apoio dos serviços sociais^(5, 6)^.

Considerando que a APS é responsável pelo cuidado inicial e contínuo, enquanto a AE atua no manejo de casos de maior complexidade, torna-se fundamental compreender como o cuidado de enfermagem se organiza em cada nível de atenção, com foco na integralidade do cuidado. Ademais, diante da escassez de estudos que abordem especificamente o papel da enfermagem no contexto da CPC em diferentes pontos da rede, a análise comparativa entre esses níveis permite identificar lacunas assistenciais, fortalecer práticas fundamentadas em evidências e promover o uso mais eficiente dos recursos disponíveis na RAS. Ainda, cabe destacar a ausência de estudos que abordem o cuidado de enfermagem direcionado às pessoas com CPC em qualquer nível de cuidado.

Assim, o presente estudo teve como objetivo analisar e comparar o cuidado de enfermagem oferecido aos usuários com CPC na APS e na AE.

## MÉTODO

### Desenho do Estudo

Trata-se de um estudo transversal analítico, seguindo as recomendações do *Strengthening the Reporting of Observational Studies in Epidemiology* (STROBE) para estudos observacionais^(7)^.

### Local, População e Critérios de Seleção

Para este estudo, foi utilizada uma amostra por conveniência de 226 profissionais de enfermagem, sendo 163 trabalhadores da APS e 63 da AE, distribuídas pelo Brasil. Considerando o aumento do número de profissionais contratados temporariamente durante a pandemia, especialmente no período mais crítico, houve impacto direto na definição da população elegível para o estudo, justificando o uso da amostra por conveniência, uma vez que a aplicação de um cálculo amostral não se mostrou factível.

Como critério de inclusão, foram definidos enfermeiros, técnicos de enfermagem e auxiliares de enfermagem que trabalharam na instituição durante a pandemia de Covid-19, por pelo menos três meses, e foram excluídos os participantes que estavam de licença e/ou afastamento e/ou férias durante este período.

### Coleta de Dados

O instrumento de coleta de dados foi inicialmente elaborado em um documento no formato Word, com base no Manual para Avaliação e Manejo de Condições Pós-Covid na Atenção Primária à Saúde do Ministério da Saúde^(8)^. As perguntas presentes no questionário autorrespondido foram elaboradas pelas pesquisadoras a partir da adaptação direta das orientações e diretrizes do manual, com foco na aplicabilidade prática para o público-alvo do estudo. As questões abordaram aspectos como a definição das CPC, manifestações clínicas, retorno às atividades, exames complementares, avaliação, manejo clínico e encaminhamento para serviços especializados. O questionário, composto por 20 perguntas fechadas com respostas do tipo sim/não ou múltipla escolha, foi então disponibilizado aos participantes por meio da plataforma Google Forms, o que assegurou praticidade e facilitou o acesso remoto à coleta de dados.

A coleta de dados foi realizada no período de 25 de maio de 2023 a 22 de janeiro de 2024, a pesquisa foi amplamente divulgada pelos Conselhos Regionais de Enfermagem e nas redes sociais. Essa medida foi adotada para tentar alcançar o máximo de profissionais possível de todas as regiões brasileiras, garantindo a mesma chance de resposta a todos eles.

### Análise e Tratamento Dos Dados

Após a finalização da coleta de dados, as respostas foram tabuladas no Excel, sendo organizadas em ordem cronológica (a partir do início da coleta de dados) e utilizadas para fins de análise estatística. Inicialmente, foi realizada uma análise descritiva dos dados, por meio de frequências absolutas e percentuais (variáveis qualitativas) de forma geral e por tipo de unidade (APS e AE).

Ainda, os dados foram transportados para o Programa SAS 9.4, onde foi realizada a análise dos dados^(9)^. Assim, as associações entre o tipo de unidade e as variáveis de interesse foram analisadas por meio do teste exato de Fisher^(10)^. Para todas as análises adotou-se um nível de significância de 5%.

As respostas não respondidas foram incluídas na análise final, porém foram consideradas, na análise dos dados, como ausentes e identificadas dentro de cada questão do formulário.

### Aspectos Éticos

O estudo foi aprovado pelo Comitê de Ética em Pesquisa (CEP) da Universidade Federal de São Carlos (UFSCar), sob o Certificado de Apresentação de Apreciação Ética (CAAE) 68157323.6.0000.5504 e o Número do Parecer 6.244.667. A coleta de dados foi iniciada após a assinatura do Termo de Consentimento Livre e Esclarecido (TCLE) pelo participante.

## RESULTADOS

Participaram do estudo 226 profissionais de enfermagem, sendo 91, 1% (205) enfermeiros. Destes, 52, 4% (98) autodecla-raram-se pessoas brancas, 46, 5% (87) autodeclararam-se pessoas negras e 1, 1% (2), amarelas, sendo que 39 não responderam às questões.

Os resultados evidenciaram que não foram identificadas diferenças estatísticas quanto ao cuidado dos profissionais de enfermagem sobre CPC de APS e AE (Tabela 1).

**Tabela 1:** Conhecimento e conceituação da CPC de profissionais de enfermagem – São Carlos, SP, Brasil, 2024.

Variável	Atenção especializada (n = 63)	Atenção primária (n = 163)	Valor-p^**^
Você já ouviu falar de condição pós-Covid?			0, 87
Não	19 (30, 6%)	48 (29, 4%)	
Sim	43 (69, 3%)	115 (71, 5%)	
Não respondeu = 1	1	0	
Você sabe conceituar condição pós-Covid?			0, 65
Não	30 (48, 4%)	72 (44, 2%)	
Sim	32 (51, 6%)	91 (55, 8%)	
Não respondeu = 1	1	0	

**Teste exato de Fisher.

Fonte: Elaborado pela autora.

Ao realizar a análise de comparação entre os profissionais oriundos dos diferentes serviços, destaca-se uma associação entre profissionais de enfermagem da APS e a referência da manifestação da tosse como sendo um sintoma comum da CPC e do déficit neurocognitivo como um sintoma grave. Também foi identificada uma associação entre ser profissional da AE e a identificação da inapetência entre as manifestações clínicas menos comuns da condição pós-Covid (Tabela 2).

**Tabela 2:** Manifestações clínicas de condição pós-Covid citadas pelos profissionais de enfermagem – São Carlos, SP, Brasil, 2024.

Variável	Atenção especializada (n = 63)	Atenção primária (n = 163)	Valor-p^**^
Você sabe quais são as manifestações clínicas mais comuns da condição pós-Covid?			0, 27
Não	17 (27%)	57 (35%)	
Sim	46 (73%)	106 (65%)	
Fadiga	38 (82, 6%)	91 (85, 8%)	0, 63
Tosse	23 (50%)	85 (80, 2%)	<0, 01
Dispneia	26 (56, 5%)	76 (71, 7%)	0, 09
Anosmia (alterações do olfato)	28 (60, 9%)	69 (65, 1%)	0, 71
Desconforto torácico	22 (47, 8%)	66 (62, 3%)	0, 11
Você sabe quais são as manifestações clínicas menos comuns da condição pós-Covid?^*^			0, 55
Não	31 (49, 2%)	72 (44, 2%)	
Sim	32 (50, 8%)	91 (55, 8%)	
Alopecia	16 (50%)	45 (49, 4%)	0, 99
Sudorese	14 (43, 7%)	45 (49, 4%)	0, 68
Insônia	13 (40, 6%)	35 (38, 5%)	0, 84
Diarreia	8 (25%)	37 (40, 7%)	0, 14
Artralgia e/ou mialgia	13 (40, 6%)	28 (30, 8%)	0, 38
Vertigem	10 (31, 2%)	31 (34, 1%)	0, 83
Tonturas	7 (21, 9%)	28 (30, 8%)	0, 37
Inapetência	14 (43, 7%)	20 (22%)	0, 02
Cefaleia	9 (28, 1%)	24 (26, 4%)	0, 82
Rinorreia	7 (21, 9%)	25 (27, 5%)	0, 64
Disgeusia (alterações do paladar)	6 (18, 7%)	16 (17, 6%)	0, 99
Você sabe quais são as manifestações clínicas neuropsiquiátricas da condição pós-Covid?			0, 65
Não	21 (33, 3%)	60 (36, 8%)	
Sim	42 (66, 7%)	103 (63, 2%)	
Perda de memória	30 (71, 4%)	82 (80, 4%)	0, 27
Redução da concentração	29 (69%)	75 (72, 8%)	0, 69
Ansiedade/depressão	27 (64, 3%)	73 (71, 6%)	0, 43
Transtorno de estresse pós-traumático	15 (35, 7%)	45 (44, 1%)	0, 46
Não respondeu = 1	0	1	
Você sabe quais são as manifestações clínicas mais graves da condição pós-Covid?			0, 30
Não	24 (38, 1%)	75 (46%)	
Sim	39 (61, 9%)	88 (54%)	
Fibrose pulmonar	26 (68, 4%)	60 (68, 2%)	0, 99
Distúrbios tromboembólicos	25 (65, 8%)	55 (62%)	0, 84
Déficit neurocognitivo	16 (42,1%)	55 (62%)	0,04
Sequela de dano miocárdico viral com redução de função sistólica e arritmias	19 (50%)	41 (46,6%)	0,85
Síndrome de Guillain-Barré	10 (26,3%)	23 (26,1%)	0,99
Rash com diferentes apresentações possíveis (vesicular, maculopapular, urticariforme ou similar a eritema pérnio)	6 (15,8%)	18 (20,4%)	0,63
Não respondeu = 1	1	0	

**Teste exato de Fisher.

Fonte: Elaborado pela autora.

Ainda, não foi identificada relação estatística entre o que foi relatado pelos profissionais da APS e os da AE quanto à infecção ativa e diagnóstico de condição pós-Covid (Tabela 3).

**Tabela 3:** Cuidados de enfermagem da AE e APS sobre infecção ativa e diagnóstico de CPC – São Carlos, SP, Brasil, 2024.

Variável	Atenção especializada (n = 63)	Atenção primária (n = 163)	Valor-p^**^
Você acha que é possível ter uma infecção viral ativa em pacientes com CPC?			0,69
Não	11 (17,7%)	25 (15,6%)	
Sim	51 (82,3%)	135 (84,4%)	
Não respondeu = 4	1	3	
Você acha que um indivíduo com CPC apresenta PCR positivo?			0,99
Não	24 (38,7%)	63 (39,6%)	
Sim	38 (61,3%)	96 (60,4%)	
Não respondeu = 5	1	4	
Você sabe como se diagnostica a CPC?			0,76
Não	36 (59%)	90 (55,9%)	
Sim	25 (41%)	71 (44,1%)	
Não respondeu = 4	2	2	
Há a indicação de retestar os pacientes com CPC com exames para avaliação de infecção ativa pelo SARS-CoV-2 (RT-PCR/RT-LAMP ou teste de antígeno), nos primeiros 90 dias após a primoinfecção?			0,76
Não	25 (41,7%)	60 (38,5%)	
Sim	35 (58,3%)	96 (61,5%)	
Não respondeu = 10	3	7	
Você sabe se casos que surgem com nova sintomatologia nos primeiros 90 dias da primoinfecção, sem outra etiologia definida, e, principalmente, se houve contato com caso confirmado, podem ser retestados conforme avaliação médica e da vigilância epidemiológica?			0,99
Não	23 (37,7%)	58 (36,7%)	
Sim	38 (62,3%)	100 (63,3%)	
Não respondeu = 7	2	5	
Se o indivíduo apresentar novos sintomas compatíveis com síndrome gripal, após os 90 dias, recomenda-se nova testagem com exames de detecção viral?			0,42
Não	8 (12,9%)	29 (18,5%)	
Sim	54 (87,1%)	128 (81,5%)	
Não respondeu = 7	1	6	

**Teste exato de Fisher.

Fonte: Elaborado pela autora.

As análises estatísticas não encontraram associação entre a unidade de origem do participante da pesquisa e as indicações da realização de exames complementares e ações assistenciais aos usuários com condição pós-Covid (Tabela 4).

**Tabela 4:** Cuidados de enfermagem na AE e APS à pessoa com CPC – São Carlos, SP, Brasil, 2024.

Variável	Atenção especializada (n = 63)	Atenção primária (n = 163)	Valor-p^**^
Há a indicação de realização de exames complementares em todos os casos de pacientes com CPC?			0,45
Não	23 (36,5%)	69 (42,3%)	
Sim	40 (63,5%)	94 (57,7%)	
Quais são as situações clínicas que exigem exames complementares para a avaliação de CPC?^*^			
Pacientes em recuperação de doença grave, com alta após hospitalização, anormalidades laboratoriais identificadas previamente ou aqueles com sintomas persistentes inexplicáveis.	34 (54%)	106 (65%)	0,13
Doença complicada por insuficiência cardíaca ou miocardite, ou com seus sinais e sintomas, como dispneia, desconforto torácico, edema.	34 (54%)	99 (60,7%)	0,37
Fadiga ou fraqueza inexplicadas	29 (46%)	69 (42,3%)	0,65
Artralgia, mialgia e outros sinais/sintomas compatíveis com doença reumatológica.	23 (36,1%)	55 (33,7%)	0,76
Recrudescência da febre.	12 (19%)	46 (28,2%)	0,18
Quais ações de cuidado são necessárias a um paciente com CPC?^*^			
Avaliação e manejo de comorbidades	52 (82,5%)	129 (79,1%)	0,71
Atenção a cuidados de saúde geral: alimentação, evitar tabagismo e álcool, qualidade do sono.	27 (42,9%)	91 (55,8%)	0,10
Atenção à saúde mental	27 (42,9%)	82 (50,3%)	0,37
Aumento gradual de exercício físico	16 (25,4%)	62 (38%)	0,09

*Admite múltiplas respostas, por isso a soma dos percentuais não será 100%

**Teste exato de Fisher

Fonte: Elaborado pela autora.

Também foi identificada uma associação entre a origem do profissional de saúde e o que foi referido sobre a necessidade de encaminhamento de casos de CPC com persistência de dispneia ou tosse após 12 semanas do início agudo da Covid-19 (Tabela 5).

**Tabela 5:** Comparação entre o serviço de origem dos profissionais de saúde e as condições de encaminhamentos de pessoas com CPC – São Carlos, SP, Brasil, 2024.

Variável	Atenção especializada (n = 63)	Atenção primária (n = 163)	Valor-p^**^
Com quais condições pacientes com CPC precisam ser encaminhados aos serviços especializados?^*^			
Suspeita ou diagnóstico de complicações como tromboembolismo, infarto agudo do miocárdio, pericardite, miocardite, insuficiência cardíaca descompensada ou evento neurológico agudo	37 (58,7%)	106 (65%)	0,44
Pacientes que apresentaram comprometimento pulmonar grave, fibrose pulmonar ou ventilação mecânica prolongada	33 (52,4%)	97 (59,5%)	0,37
Paciente com história de tromboembolismo pulmonar pós-covid que permanece com dispneia, cansaço, intolerância ao exercício, tontura e/ou síncope após 12 semanas do evento tromboembólico	34 (54%)	87 (53,4%)	0,99
Sintomas respiratórios persistentes após 12 semanas	30 (47,6%)	78 (47,8%)	0,99
Dispneia ou tosse persistente e sem resposta ao tratamento clínico otimizado após 12 semanas do quadro agudo da Covid-19	19 (30,2%)	77 (47,2%)	0,02
Síndrome coronariana aguda em contexto de infecção por covid-19	24 (38,1%)	70 (42,9%)	0,55
Depressão, ansiedade e/ ou transtorno de estresse pós-traumático	27 (42,9%)	63 (38,6%)	0,65
Suspeita de miocardite	25 (39,7%)	63 (38,6%)	0,88
Novo diagnóstico de insuficiência cardíaca ou piora sintomática de insuficiência cardíaca preexistente	22 (34,9%)	63 (38,6%)	0,65
Pacientes com quadros leves a moderados de covid-19 que permanecem com dispneia após 12 semanas do quadro inicial	22 (34,9%)	55 (33,7%)	0,88
Risco de suicídio ou de heteroagressão, risco de exposição moral e sintomas psicóticos agudizados	24 (38,1%)	51 (31,3%)	0,35
Necessidade de manter oxigenioterapia após 4 semanas da alta hospitalar	16 (25,4%)	59 (36,2%)	0,16
Pericardite, após avaliação na emergência, se sintomas persistentes após tratamento adequado com anti-inflamatórios não esteroides	19 (30,2%)	53 (32,5%)	0,87
Declínio cognitivo e/ou polineuropatia	16 (25,4%)	54 (33,1%)	0,34
Palpitação recorrente de origem indeterminada	13 (20,6%)	53 (32,5%)	0,10
Taquicardia supraventricular sintomática ou recorrente	15 (23,8%)	51 (31,3%)	0,33
Fibrilação atrial com possibilidade de cardioversão	15 (23,8%)	47 (28,8%)	0,51
Suspeita de síndrome da taquicardia postural ortostática	11 (17,5%)	46 (28,2%)	0,12
Flutter atrial	9 (14,3%)	41 (25,1%)	0,11

*Admite múltiplas respostas, por isso a soma dos percentuais não será 100%

**Teste exato de Fisher.

Fonte: Elaborado pela autora.

## DISCUSSÃO

Os resultados indicam uma lacuna no cuidado de enfermeiros da AE e APS sobre a CPC, que pode estar relacionada ao fato de ser uma doença nova ou à complexidade de identificação, além da falta de capacitação disponibilizada pelos serviços e ausência da implementação efetiva das diretrizes direcionadas ao cuidado da doença.

Nesse cenário, a identificação da CPC nos serviços de saúde é dificultada por seu caráter sistêmico e pela variabilidade dos sintomas, que muitas vezes diferem daqueles observados na fase aguda da infecção por SARS-CoV-2^(11, 12)^. Os sintomas mais comuns da CPC são fadiga, intolerância ao esforço, dispneia e mialgia, enquanto manifestações menos frequentes incluem diarreia, dor abdominal, anorexia, alterações do olfato e do paladar, dor de garganta, ganho ou perda de peso, tosse, palpitação e desconforto torácico^(13)^.

A persistência desses sintomas pode estar relacionada aos sintomas respiratórios agudos e à hospitalização prolongada durante a fase aguda da Covid-19. As formas graves da Covid-19, que frequentemente causam hospitalizações prolongadas, podem provocar respostas imunológicas intensas e danos a órgãos. Além disso, tratamentos agressivos, intubação e infecções nosocomiais associadas ao manejo de sintomas respiratórios agudos também podem contribuir para sinais e sintomas persistentes^(13)^. Ainda, a semelhança com outras condições pós-infecciosas, como a encefalomielite miálgica/síndrome da fadiga crônica e a fibromialgia implicam na definição do diagnóstico de CPC^(14)^.

É importante destacar que a identificação dos sintomas da CPC pode variar significativamente entre os diferentes níveis de atenção à saúde. Os profissionais da APS mantêm contato contínuo e direto com os pacientes em seu território de abrangência, seja por meio de consultas regulares ou visitas domiciliares. Essa proximidade com o usuário favorece a identificação precoce e recorrente de sintomas mais comuns, como tosse persistente, sendo frequentemente relatados pelos pacientes e facilmente observados pelos profissionais de enfermagem, especialmente em contextos de vigilância pós-infecção^(15, 16)^.

No entanto, déficits neurocognitivos, como dificuldade de concentração, névoa mental e perda de memória, podem ser interpretados como manifestações mais graves devido ao seu impacto direto na funcionalidade e qualidade de vida, interferindo na vida cotidiana. Soma-se a isso uma crescente divulgação na literatura sobre as sequelas neurológicas associadas à infecção por Covid-19, o que pode contribuir para a ampliação dessa compreensão da gravidade dos sintomas^(15, 16)^.

Por outro lado, a AE é mais centrada em atendimentos pontuais, predominantemente direcionados para casos de maior complexidade clínica, com foco em agravos específicos que demandam intervenções diagnósticas e terapêuticas mais direcionadas. Nesse contexto, sintomas inespecíficos tendem a ser subvalorizados ou não priorizados durante a consulta, em comparação com manifestações mais evidentes ou incapacitantes^(17)^. Deste modo, diferenças na abordagem clínica e vínculo dos enfermeiros entre APS e AE influenciam na identificação da gravidade dos sintomas da CPC, com a APS favorecendo a observação de alterações sutis e a AE focada em casos mais complexos^(18, 19)^.

No contexto da APS, especialmente, o enfermeiro desempenha um papel multidimensional ao prestar assistência e gerenciar o cuidado em contato direto com os usuários. Entretanto, esses profissionais enfrentam desafios significativos na implementação de diretrizes, acesso facilitado aos serviços de saúde e a realização de acolhimento direcionado^(20)^. Ainda, quando se deparam com casos clínicos de CPC que não podem ser resolvidos na APS e necessitam ser encaminhados para níveis especializados de atenção à saúde, também enfrentam dificuldades associadas à incerteza clínica, que apresenta, como consequência, a prestação fragmentada de cuidados e mal coordenada e a falta de abordagens multidisciplinares, negligenciando as necessidades psicossociais dos usuários e focando no modelo biomédico^(21)^.

Neste contexto, a falta de integração entre os níveis de atenção, aliada à ausência de fluxos claros de referência e contrarreferência, contribui para o atraso nos diagnósticos e a sobrecarga da AE. Nesse sentido, propõe-se a adoção de estratégias como o uso de prontuários eletrônicos, a padronização de critérios clínicos para estratificação de risco e a criação de ambulatórios especializados para facilitar a identificação precoce e o encaminhamento adequado dos casos. Ainda, a devolutiva de informações clínicas à APS por meio da contrarreferência é igualmente importante para garantir a continuidade e resolutividade do cuidado^(22)^.

No cenário brasileiro, há notável dificuldade na regulação entre APS e AE, com uma longa fila de espera para consultas e exames ocasionada pela limitada resolutividade das demandas na APS. Além disso, a subnotificação de sintomas nos prontuários médicos tem sido uma questão recorrente, que pode ocorrer devido à falta de comunicação adequada por parte dos usuários ou à descrição inadequada pelos profissionais de saúde. Desse modo, destaca-se a necessidade de abordagens integradas e multidisciplinares na APS, especialmente para lidar com as complexas CPC^(16)^.

Em um estudo realizado na Bélgica, os profissionais da APS referem que o acesso facilitado a materiais da literatura científica ou de protocolos de manejo à CPC é insuficiente e limitado, sendo o tempo de duração dos sinais e sintomas o critério diagnóstico mais comum para identificação dos casos. Além disso, o conhecimento insuficiente do cuidado e da identificação de casos da CPC no serviço está relacionado à ausência de treinamentos ou informações por parte da instituição de trabalho ou ensino sobre a doença^(23)^.

No Brasil, destaca-se a existência do Manual para avaliação e manejo de condições pós-Covid na Atenção Primária à Saúde, desenvolvido pelo Ministério da Saúde; entretanto, verifica-se uma falta de divulgação deste material nos serviços, em vista das dificuldades dos profissionais de enfermagem em identificar as manifestações clínicas comuns à doença, manejo e critérios de encaminhamento ao serviço especializado^(8)^. Nesse contexto, a publicação de um material não garante o alcance dos profissionais ou que eles o utilizem de maneira adequada, uma vez que a ausência de programas de estratégias em educação permanente dificulta o acesso e influi na aplicação adequada dessas diretrizes^(20)^.

Posto isso, destaca-se que o desconhecimento sobre as manifestações clínicas e a complexidade da CPC entre profissionais de enfermagem compromete a capacidade de detecção precoce, acompanhamento adequado e manejo clínico apropriado dos pacientes acometidos. Dada a natureza multifatorial e frequentemente inespecífica dos sintomas, a ausência de conhecimento específico pode levar à subvalorização das queixas, atrasos no diagnóstico e falhas na condução de casos que requerem cuidado contínuo. Tal lacuna impacta negativamente a qualidade da assistência, especialmente na APS, onde a enfermagem atua como ponto de contato inicial e elemento central na coordenação do cuidado^(17,18,19)^.

Portanto, enfatiza-se a necessidade do investimento na formação continuada da enfermagem e na incorporação do manual do Ministério da Saúde sobre as CPC na rotina assistencial na APS, que pode ser operacionalizada por meio do uso de *checklists* clínicos padronizados. Dessa forma, o uso sistemático do referido manual, aliado à integração entre APS e AE, constitui uma estratégia estruturante para qualificar o cuidado às pessoas com CPC no sistema de saúde brasileiro^(22)^.

Consequentemente, o desafio do cuidado de enfermagem não se limita ao relacionamento enfermeiro-usuário, mas também envolve o cuidado e a adaptação dos próprios profissionais. Ainda, a colaboração interprofissional e o envolvimento ativo dos usuários em seus cuidados também são essenciais, mas há uma considerável falta de especialistas na área, além da limitação de atividades de educação permanente planejadas pelos gestores, especialmente no nível municipal^(20, 24)^.

De maneira geral, a atuação da equipe de enfermagem em todos os níveis de cuidado é considerada fundamental para oferecer um cuidado integral aos usuários com CPC, incluindo ações para identificação e monitoramento de usuários, orientações e suporte, realização de atividades de educação em saúde, participação de pesquisas clínicas e coordenação do cuidado de forma multiprofissional, junto de clínicos gerais, médicos especialistas e fisioterapeutas^(24)^.

No contexto da APS, as necessidades de saúde identificadas pela equipe de enfermagem devem ser realizadas de forma planejada, incluindo consultas domiciliares. Além disso, os profissionais devem apoiar os cuidadores no manejo da CPC e de outras comorbidades adjacentes. Ademais, estimular a criação de grupos de educação em saúde para usuários com CPC contribui para o autocuidado e conhecimento dos sintomas prolongados da Covid-19^(25)^.

Por outro lado, a AE, que abrange serviços de tecnologias de média complexidade, deve ter uma equipe multidisciplinar (psicólogo, enfermeiro, fisioterapeuta, nutricionista e terapeuta ocupacional) para propiciar a realização de exames, testes diagnósticos e estratégias terapêuticas de manejo dos sintomas da CPC. Assim, as competências da AE incluem acolher o usuário, compreender suas necessidades e realizar avaliações multiprofissionais de forma integral, idealmente por meio de um Projeto Terapêutico Singular (PTS) para identificar cuidados individualizados. Além disso, o cuidado aos acometidos com CPC deve ser compartilhado com a APS, considerando a evolução clínica e as necessidades dos usuários, e os casos graves devem ser encaminhados a serviços de emergência^(26,27)^.

Dessa forma, o referenciamento deve ser realizado considerando fatores biopsicossociais, de forma a contemplar a multi-disciplinaridade, sendo necessário que o profissional de enfermagem, além de reconhecer qualquer encaminhamento, desenvolva um plano de reabilitação e gestão de saúde personalizado para cada indivíduo, considerando a família e a comunidade^(25,28)^.

Nesse sentido, há a necessidade de elaboração de protocolos assistenciais para os serviços de saúde (em especial da AE, uma vez que há produção científica escassa acerca do manejo da condição pós-Covid neste nível de atenção à saúde), visando facilitar a comunicação entre os profissionais e os níveis de cuidado, além de assegurar a qualidade na tomada de decisões, prevenindo riscos e promovendo inovações nos cuidados de enfermagem^(29)^.

Este estudo evidencia a necessidade do desenvolvimento de estratégias para a implementação de protocolos já existentes e novos, que visem organizar o manejo clínico e os critérios de encaminhamento da CPC, diante da escassez na literatura. Além disso, destaca-se a necessidade de a equipe de enfermagem reconhecer-se como coordenadora do cuidado aos acometidos pela CPC e criadora de planos de cuidados individualizados, com ênfase no manejo biopsicossocial do usuário, bem como a necessidade urgente do fortalecimento das políticas de educação permanente, especialmente em nível municipal.

Por fim, considera-se como limitação deste estudo a baixa adesão dos profissionais de enfermagem à pesquisa, mesmo sendo realizada em âmbito nacional e sendo amplamente divulgada, especialmente pelo conselho de classe. Ainda, o estudo explorou o conhecimento dos enfermeiros por meio do autorrelato destes profissionais, o que pode não refletir com precisão o conhecimento técnico real, uma vez que está sujeito a viés de memória, desejabilidade social ou superestimação do conhecimento e da prática clínica dos respondentes. Além disso, o uso de amostragem por conveniência pode ter restringido a representatividade dos participantes, limitando a generalização dos resultados. Apesar disso, o estudo apresenta resultados robustos, contribuindo significativamente para a compreensão dos desafios enfrentados pelos profissionais de enfermagem na identificação e manejo da CPC, reforçando a necessidade de estratégias estruturadas de capacitação e organização do cuidado.

## CONCLUSÃO

Os resultados deste estudo indicam diferenças pontuais no que foi referido pelos enfermeiros da APS e AE, especialmente quanto à identificação de manifestações clínicas específicas e à necessidade de encaminhamento em casos de sintomas persistentes. No entanto, não foi verificada diferença significativa nas respostas obtidas entre os profissionais dos diferentes níveis de cuidado em relação ao diagnóstico, exames complementares ou ações assistenciais.

Ainda, ao analisar os resultados, verifica-se um conhecimento “básico” sobre a doença (isto é, conceituações e manifestações comuns e de fácil designação) e o seu manejo, diagnóstico e identificação, uma vez que as respostas contemplam o cuidado e reconhecimento da Covid-19, mas não necessariamente da CPC. Ademais, a falta de conhecimento sobre a CPC faz-se presente, uma vez que muitos profissionais de enfermagem enfrentam dificuldades em reconhecer os sintomas, que podem ser confundidos com outras condições.

Deste modo, a identificação e o manejo clínico da CPC são prejudicados pela ausência de protocolos amplamente divulgados, pela ausência e insuficiência de capacitação dos profissionais de enfermagem e pela frágil integração entre os níveis de atenção à saúde. Esses fatores comprometem a qualidade do cuidado prestado, tanto na APS quanto na AE, favorecendo abordagens fragmentadas e dificultando a resposta efetiva às necessidades dos usuários.

Os achados deste estudo ressaltam o papel central da enfermagem na coordenação do cuidado às pessoas com CPC, evidenciando a urgência no desenvolvimento de estratégias de educação permanente e capacitação contínua para a equipe de enfermagem, com foco específico na CPC. Isso pode garantir a implementação efetiva de fluxos de encaminhamento, promovendo um cuidado mais integrado e eficaz.

Além disso, as atuações integradas e multidisciplinares, bem como o fortalecimento da APS como porta de entrada dos serviços de saúde, são caminhos fundamentais para aprimorar a excelência do cuidado e atender às necessidades dos usuários. Portanto, este estudo contribui para futuras pesquisas e o desenvolvimento de políticas públicas que busquem garantir um cuidado de qualidade, integrado e acessível aos acometidos pela CPC no Brasil.

## DISPONIBILIDADE DE DADOS

Todo o conjunto de dados que dá suporte aos resultados deste estudo foi publicado no próprio artigo.

# Translation: Atención de enfermería a usuarios con condición post-COVID en la atención primaria y especializada de salud

**DOI:** 10.1590/1980-220x-reeusp-2025-0043es

**Authors:** Rafaela Deharo Curvelo, Ana Cristina Ribeiro La Scaléa, Sílvia Carla da Silva André Uehara

**Affiliations:** 1 Universidade Federal de São Carlos, Centro de Ciências Biológicas e da Saúde, Departamento de Enfermagem,São Carlos, SP, Brasil.

**Keywords:** Enfermeras Practicantes, Síndrome Post Agudo de COVID-19, Atención Primaria de Salud, Atención Secundaria de Salud

## Abstract

**Objetivo:**

Analizar y comparar la atención de enfermería prestada a los usuarios con condición post-covid (CPC) en la atención primaria de salud (APS) y la atención especializada (AE).

**Método:**

Se trata de un estudio transversal analítico realizado con 226 profesionales de enfermería de la APS y la AE de Brasil. Los datos se recopilaron mediante un formulario autoadministrado difundido por los Consejos de Enfermería y en las redes sociales, y se analizaron mediante estadística descriptiva, seguida de la prueba t para la comparación de medias y pruebas de chi cuadrado para el estudio de la asociación con datos categóricos. El estudio fue aprobado por el Comité de Ética en Investigación de la UFSCar.

**Resultados:**

Cabe destacar una asociación entre los profesionales de enfermería de la APS y la identificación de la tos persistente como el síntoma más común de la CPC (80.2% frente a 50%; p < 0, 01) y del déficit neurocognitivo como síntoma más grave (62% frente a 42, 1%; p = 0, 04); y una asociación entre los profesionales de la AE y la identificación de la inapetencia como el síntoma menos común del CPC (43, 7% frente a 22%; p = 0, 02).

**Conclusión:**

Los resultados ponen de manifiesto retos como la dificultad para identificar la CPC, la ausencia de protocolos ampliamente utilizados y la formación limitada de los profesionales de enfermería de APS y AE, lo que repercute en la calidad de la atención.

## INTRODUCCIÓN

La condición post-covid (CPC) o COVID prolongado ha sido definida por la Organización Mundial de la Salud (OMS) como la presencia de signos y síntomas persistentes que afectan a las personas tras la infección aguda por COVID-19 en entre el 10% y el 20% de las personas que se han recuperado de la enfer-medad^(1)^. En este estudio, se está considerando el concepto de “condición post-COVID” (CPC), definido en la nota técnica n.º 57/2023 del Ministerio de Salud como la presencia de “signos, síntomas y/o condiciones que continúan o se desarrollan cuatro semanas o más después de la infección inicial por SARS-CoV-2 y que no pueden justificarse por un diagnóstico alternativo”^(2)^.

En este contexto, la CPC es una inflamación crónica sistémica que puede presentar síntomas como fatiga, disnea, afecciones neurológicas y psiquiátricas, tos, dolores musculares y articulares, alteraciones del olfato y el gusto, además de otras manifestaciones clínicas multifactoriales, que pueden mejorar, agravarse o reaparecer con el tiempo. Así, al ser una afección compleja e incapacitante que afecta a las actividades diarias debido a la diversidad de síntomas, con una etiología poco clara, se requiere sensibilidad para realizar un diagnóstico y un manejo adecuados, lo que aumenta los retos para los servicios de salud^(2)^.

Para ello, es esencial un enfoque integrado y multidisciplinario, con un plan de cuidados completo e individualizado. Así, la Atención Primaria de Salud (APS), considerada la principal puerta de entrada al Sistema Único de Salud (SUS), realiza la evaluación y el manejo inicial de los casos de CPC con el fin de garantizar una atención continua y integral. Además, los casos de mayor complejidad deben ser derivados a la Atención Especializada (AE), optimizando los recursos de la Red de Atención de Salud (RAS) y abordando las afecciones crónicas y graves de manera eficiente^(3)^.

Debido a esta heterogeneidad, la enfermería, al comprender la clase de profesionales en contacto constante con los usuarios de los servicios de salud, tiene un papel esencial en la acogida y el cuidado de las personas con CPC, ya que debe integrar acciones de educación en salud, asistencia directa y coordinación con redes de apoyo social^(4)^. Como estrategia de manejo de la CPC, se recomienda la rehabilitación física como profilaxis y tratamiento directo, el monitoreo de las comorbilidades preexistentes para evitar agravamientos y factores de riesgo para el desarrollo de la afección, ofrecer apoyo a la salud mental y, entre aquellos que enfrentan dificultades sociales y financieras, ofrecer apoyo de los servicios sociales^(5, 6)^.

Teniendo en cuenta que la APS es responsable de la atención inicial y continua, mientras que la AE actúa en el manejo de casos de mayor complejidad, es fundamental comprender cómo se organiza la atención de enfermería en cada nivel de atención, con un enfoque en la integralidad de la atención. Además, dada la escasez de estudios que aborden específicamente el papel de la enfermería en el contexto de la CPC en diferentes puntos de la red, el análisis comparativo entre estos niveles permite identificar las lagunas asistenciales, fortalecer las prácticas basadas en la evidencia y promover un uso más eficiente de los recursos disponibles en la Red de Atención a la Salud (RAS). Además, cabe destacar la ausencia de estudios que aborden la atención de enfermería dirigida a las personas con CPC en cualquier nivel de atención.

Por lo tanto, el presente estudio tuvo como objetivo analizar y comparar la atención de enfermería ofrecida a los usuarios con CPC en la APS y en la AE.

## MÉTODO

### Diseño Del Estudio

Se trata de un estudio transversal analítico, siguiendo las recomendaciones del Strengthening the Reporting of Observational Studies in Epidemiology (STROBE) para estudios observacionales^(7)^.

### Lugar, Población y Criterios de Selección

Para este estudio, se utilizó una muestra por conveniencia de 226 profesionales de enfermería, de los cuales 163 trabajaban en la APS y 63 en la AE, distribuidos por todo Brasil. Teniendo en cuenta el aumento del número de profesionales contratados temporalmente durante la pandemia, especialmente en el período más crítico, hubo un impacto directo en la definición de la población elegible para el estudio, lo que justificó el uso de una muestra por conveniencia, ya que la aplicación de un cálculo muestral no resultó factible.

Como criterio de inclusión, se definieron enfermeros, técnicos de enfermería y auxiliares de enfermería que trabajaron en la institución durante la pandemia de COVID-19 durante al menos tres meses, y se excluyó a los participantes que estaban de baja y/o excedencia y/o vacaciones durante este periodo.

### Recolección de Datos

El instrumento de recolección de datos se elaboró inicialmente en un documento en formato Word, basándose en el Manual para la Evaluación y el Manejo de las Afecciones Post-COVID en la Atención Primaria de Salud del Ministerio de Salud^(8)^. Las preguntas del cuestionario autoadministrado fueron elaboradas por las investigadoras a partir de la adaptación directa de las orientaciones y directrices del manual, centrándose en la aplicabilidad práctica para el público objetivo del estudio. Las preguntas abordaron aspectos como la definición de las CPC, las manifestaciones clínicas, el regreso a las actividades, los exámenes complementarios, la evaluación, el manejo clínico y la derivación a servicios especializados. El cuestionario, compuesto por 20 preguntas cerradas con respuestas de tipo sí/no o de opción múltiple, se puso a disposición de los participantes a través de la plataforma Google Forms, lo que garantizó la practicidad y facilitó el acceso remoto a la recopilación de datos.

La recolección de datos se llevó a cabo entre el 25 de mayo de 2023 y el 22 de enero de 2024, y la encuesta fue ampliamente difundida por los Consejos Regionales de Enfermería y en las redes sociales. Esta medida se adoptó para intentar llegar al mayor número posible de profesionales de todas las regiones brasileñas, garantizando las mismas oportunidades de respuesta a todos ellos.

### Análisis y Tratamiento de Los Datos

Una vez finalizada la recolección de datos, las respuestas se tabularon en Excel, se organizaron en orden cronológico (desde el inicio de la recopilación de datos) y se utilizaron para fines de análisis estadístico. Inicialmente, se realizó un análisis descriptivo de los datos, mediante frecuencias absolutas y porcentuales (variables cualitativas) en general y por tipo de unidad (APS y AE).

Además, los datos se transfirieron al programa SAS 9.4, donde se realizó el análisis de los datos^(9)^. Así, las asociaciones entre el tipo de unidad y las variables de interés se analizaron mediante la prueba exacta de Fisher^(10)^. Para todos los análisis se adoptó un nivel de significación del 5%.

Las respuestas no contestadas se incluyeron en el análisis final, pero se consideraron, en el análisis de los datos, como ausentes y se identificaron dentro de cada pregunta del formulario.

### Aspectos Éticos

El estudio fue aprobado por el Comité de Ética en Investigación (CEP) de la Universidad Federal de São Carlos (UFSCar), bajo el Certificado de Presentación de Apreciación Ética (CAAE) 68157323.6.0000.5504 y el Número de Dictamen 6.244.667. La recopilación de datos se inició tras la firma del Término de Consentimiento Libre e Ilustrado (TCLE) por parte del participante.

## RESULTADOS

Participaron en el estudio 226 profesionales de enfermería, de los cuales el 91.1% (205) eran enfermeros. De ellos, el 52.4% (98) se autodeclararon blancos, el 46.5% (87) se autodeclararon negros y el 1.1% (2) amarillos, mientras que 39 no respondieron a las preguntas.

Los resultados evidenciaron que no se identificaron diferencias estadísticas en cuanto a la atención de los profesionales de enfermería sobre la CPC de APS y AE (Tabla 1).

**Tabla 1:** Conocimiento y conceptualización de la APS por los profesionales de enfermería – São Carlos, SP, Brasil, 2024.

Variable	Atención especializada (n = 63)	Atención primaria (n = 163)	p-valor^**^
¿Ha oído hablar alguna vez de una condición post-covid?			0, 87
No	19 (30,6%)	48 (29,4%)	
Sí	43 (69,3%)	115 (71,5%)	
Sin respuesta = 1	1	0	
¿Sabe cómo conceptualizar una condición post-covid?			0, 65
No	30 (48,4%)	72 (44,2%)	
Sí	32 (51,6%)	91 (55,8%)	
Sin respuesta = 1	1	0	

**Prueba exacta de Fisher.

Fuente: Elaboración propia.

Al analizar la comparación entre los profesionales de los diferentes servicios, se observó una asociación entre los profesionales de enfermería de APS y la referencia a la tos como síntoma común de la CPC y al déficit neurocognitivo como síntoma grave. También se identificó una asociación entre ser profesional de APS e identificar la inapetencia entre las manifestaciones clínicas menos frecuentes de la condición post-covid (Tabla 2).

**Tabla 2:** Manifestaciones clínicas de la condición post-covid citadas por los profesionales de enfermería – São Carlos, SP, Brasil, 2024.

Variable	Atención especializada (n = 63)	Atención primaria (n = 163)	p-valor^**^
¿Sabe cuáles son las manifestaciones clínicas más frecuentes de la condición post-covid?			0,27
No	17 (27%)	57 (35%)	
Sí	46 (73%)	106 (65%)	
Fatiga	38 (82,6%)	91 (85,8%)	0,63
Tos	23 (50%)	85 (80,2%)	< 0,01
Disnea	26 (56,5%)	76 (71,7%)	0,09
Anosmia (alteración del sentido del olfato)	28 (60,9%)	69 (65,1%)	0,71
Malestar torácico	22 (47,8%)	66 (62,3%)	0,11
¿Sabe cuáles son las manifestaciones clínicas menos frecuentes de la condición post-covid?			0,55
No	31 (49,2%)	72 (44,2%)	
Sí	32 (50,8%)	91 (55,8%)	
Alopecia	16 (50%)	45 (49,4%)	0,99
Sudoración	14 (43,7%)	45 (49,4%)	0,68
Insomnio	13 (40,6%)	35 (38,5%)	0,84
Diarrea	8 (25%)	37 (40,7%)	0,14
Artralgia y/o mialgia	13 (40,6%)	28 (30,8%)	0,38
Vértigo	10 (31,2%)	31 (34,1%)	0,83
Mareo	7 (21,9%)	28 (30,8%)	0,37
Inapetencia	14 (43,7%)	20 (22%)	0,02
Dolor de cabeza	9 (28,1%)	24 (26,4%)	0,82
Rinorrea	7 (21,9%)	25 (27,5%)	0,64
Disgeusia (cambios en el gusto)	6 (18,7%)	16 (17,6%)	0,99
¿Sabe cuáles son las manifestaciones clínicas neuropsiquiátricas de la condición post-covid?			0,65
No	21 (33,3%)	60 (36,8%)	
Sí	42 (66,7%)	103 (63,2%)	
Pérdida de memoria	30 (71,4%)	82 (80,4%)	0,27
Concentración reducida	29 (69%)	75 (72,8%)	0,69
Ansiedad/depresión	27 (64,3%)	73 (71,6%)	0,43
Trastorno de estrés postraumático	15 (35,7%)	45 (44,1%)	0,46
Sin respuesta = 1	0	1	
¿Sabe cuáles son las manifestaciones clínicas más graves de la condición post-covid?			0,30
No	24 (38,1%)	75 (46%)	
Sí	39 (61,9%)	88 (54%)	
Fibrosis pulmonar	26 (68,4%)	60 (68,2%)	0,99
Trastornos tromboembólicos	25 (65,8%)	55 (62%)	0,84
Déficit neurocognitivo	16 (42,1%)	55 (62%)	0,04
Secuelas de daño miocárdico vírico con reducción de la función sistólica y arritmias	19 (50%)	41 (46,6%)	0,85
Síndrome de Guillain-Barré	10 (26,3%)	23 (26,1%)	0,99
Erupción con diferentes presentaciones posibles (vesicular, maculopapular, urticarial o similar al eritema pectoris)	6 (15,8%)	18 (20,4%)	0,63
Sin respuesta = 1	1	0	

**Prueba exacta de Fisher.

Fuente: Elaboración propia.

Además, no fue identificada relación estadística entre lo relatado por los profesionales de APS y CS sobre infección activa y diagnóstico de condición post-covid (Tabla 3).

**Tabla 3:** Cuidados de enfermería prestados por SC y APS sobre infección activa y diagnóstico de CPC – São Carlos, SP, Brasil, 2024.

Variable	Atención especializada (n = 63)	Atención primaria (n = 163)	p-valor^**^
¿Cree que es posible tener una infección vírica activa en pacientes con CPC?			0,69
No	11 (17,7%)	25 (15,6%)	
Sí	51 (82,3%)	135 (84,4%)	
Sin respuesta = 4	1	3	
¿Cree que una persona con CPC tiene una PCR positiva?			0,99
No	24 (38,7%)	63 (39,6%)	
Sí	38 (61,3%)	96 (60,4%)	
Sin respuesta = 5	1	4	
¿Sabe cómo se diagnostica el CPC?			0,76
No	36 (59%)	90 (55,9%)	
Sí	25 (41%)	71 (44,1%)	
Sin respuesta = 4	2	2	
¿Existe alguna indicación para volver a someter a los pacientes con CPC a pruebas para evaluar la infección activa por SRAS-CoV-2 (RT-PCR/RT-LAMP o prueba de antígenos) en los primeros 90 días tras la infección primaria?			0,76
No	25 (41,7%)	60 (38,5%)	
Sí	35 (58,3%)	96 (61,5%)	
Sin respuesta = 10	3	7	
¿Sabe si los casos que aparecen con nuevos síntomas en los primeros 90 días de la infección primaria, sin otra etiología definida, y especialmente si ha habido contacto con un caso confirmado, pueden volver a someterse a la prueba según la evaluación médica y de vigilancia epidemiológica?			0,99
No	23 (37,7%)	58 (36,7%)	
Sí	38 (62,3%)	100 (63,3%)	
Sin respuesta = 7	2	5	
Si el individuo presenta nuevos síntomas compatibles con síndrome pseudogripal pasados 90 días, ¿se recomienda repetir la prueba con pruebas de detección viral?			0,42
No	8 (12,9%)	29 (18,5%)	
Sí	54 (87,1%)	128 (81,5%)	
Sin respuesta = 7	1	6	

**Prueba exacta de Fisher.

Fuente: Elaboración propia.

Los análisis estadísticos no encontraron asociación entre la unidad de procedencia del participante en la investigación y las indicaciones para la realización de pruebas complementarias y acciones de cuidados a usuarios con condición post-covid (Tabla 4).

**Tabla 4:** Cuidados de enfermería en AE y APS para personas con CPC – São Carlos, SP, Brasil, 2024.

Variable	Atención especializada (n = 63)	Atención primaria (n = 163)	p-valor^**^
¿Están indicadas las pruebas complementarias en todos los casos de pacientes con CPC?			0, 45
No	23 (36, 5%)	69 (42, 3%)	
Sí	40 (63, 5%)	94 (57, 7%)	
¿Cuáles son las situaciones clínicas que requieren pruebas complementarias para la evaluación de la CPC?^*^.			
Pacientes que se recuperan de una enfermedad grave, dados de alta tras hospitalización, anomalías de laboratorio previamente identificadas o aquellos con síntomas persistentes inexplicables.	34 (54%)	106 (65%)	0, 13
Enfermedad complicada por insuficiencia cardiaca o miocarditis, o con sus signos y síntomas, como disnea, molestias torácicas, edema.	34 (54%)	99 (60, 7%)	0, 37
Fatiga o debilidad inexplicables	29 (46%)	69 (42, 3%)	0, 65
Artralgias, mialgias y otros signos/síntomas compatibles con enfermedad reumatológica	23 (36, 1%)	55 (33, 7%)	0, 76
Recurrencia de la fiebre.	12 (19%)	46 (28, 2%)	0, 18
¿Qué acciones asistenciales son necesarias para un paciente con CPC?^*^.			
Evaluación y manejo de comorbilidades	52 (82, 5%)	129 (79, 1%)	0, 71
Atención a los cuidados generales de salud: dieta, evitar el tabaco y el alcohol, calidad del sueño.	27 (42, 9%)	91 (55, 8%)	0, 10
Atención a la salud mental	27 (42, 9%)	82 (50, 3%)	0, 37
Aumento gradual del ejercicio físico	16 (25, 4%)	62 (38%)	0, 09

*Admite respuestas múltiples, por lo que la suma de los porcentajes no será 100%.

**Prueba exacta de Fisher.

Fuente: Elaboración propia.

También se identificó una asociación entre la procedencia del profesional sanitario y lo manifestado sobre la necesidad de derivar los casos de CPC con disnea o tos persistente 12 semanas después del inicio agudo de COVID-19 (Tabla 5).

**Tabla 5:** Comparación entre el servicio de origen de los profesionales de salud y las condiciones de derivación de las personas con CPC – São Carlos, SP, Brasil, 2024.

Variable	Atención especializada (n = 63)	Atención primaria (n = 163)	p-valor^**^
¿En qué condiciones es necesario derivar a los pacientes con CPC a los servicios especializados?^*^	,		
Sospecha o diagnóstico de complicaciones como tromboembolismo, infarto agudo de miocardio, pericarditis, miocarditis, insuficiencia cardiaca descompensada o evento neurológico agudo	37 (58,7%)	106 (65%)	0,44
Pacientes con insuficiencia pulmonar grave, fibrosis pulmonar o ventilación mecánica prolongada	33 (52,4%)	97 (59,5%)	0,37
Pacientes con antecedentes de tromboembolia pulmonar post-covid que siguen padeciendo disnea, cansancio, intolerancia al ejercicio, mareos y/o síncope 12 semanas después del evento tromboembólico	34 (54%)	87 (53,4%)	0,99
Síntomas respiratorios persistentes después de 12 semanas	30 (47,6%)	78 (47,8%)	0,99
Disnea o tos persistentes y sin respuesta al tratamiento clínico optimizado tras 12 semanas de síntomas agudos COVID-19	19 (30,2%)	77 (47,2%)	0,02
Síndrome coronario agudo en el contexto de la infección por COVID-19	24 (38,1%)	70 (42,9%)	0,55
Depresión, ansiedad y/o trastorno de estrés postraumático	27 (42,9%)	63 (38,6%)	0,65
Sospecha de miocarditis	25 (39,7%)	63 (38,6%)	0,88
Nuevo diagnóstico de insuficiencia cardiaca o empeoramiento sintomático de insuficiencia cardiaca preexistente	22 (34,9%)	63 (38,6%)	0,65
Pacientes con COVID-19 leve a moderada que siguen padeciendo disnea 12 semanas después de la afección inicial	22 (34,9%)	55 (33,7%)	0,88
Riesgo de suicidio o heteroagresión, riesgo de exposición moral y síntomas psicóticos agudos	24 (38,1%)	51 (31,3%)	0,35
Necesidad de mantener la oxigenoterapia 4 semanas después del alta hospitalaria	16 (25,4%)	59 (36,2%)	0,16
Pericarditis, tras evaluación en urgencias, si persisten los síntomas tras tratamiento adecuado con antiinflamatorios no esteroideos	19 (30,2%)	53 (32,5%)	0,87
Deterioro cognitivo y/o polineuropatía	16 (25,4%)	54 (33,1%)	0,34
Palpitaciones recurrentes de origen indeterminado	13 (20,6%)	53 (32,5%)	0,10
Taquicardia supraventricular sintomática o recurrente	15 (23,8%)	51 (31,3%)	0,33
Fibrilación auricular con posibilidad de cardioversión	15 (23,8%)	47 (28,8%)	0,51
Sospecha de síndrome de taquicardia ortostática postural	11 (17,5%)	46 (28,2%)	0,12
Flutter auricular	9 (14,3%)	41 (25,1%)	0,11

*Admite respuestas múltiples, por lo que la suma de los porcentajes no será 100%.

**Prueba exacta de Fisher.

Fuente: Elaboración propia.

## DISCUSIÓN

Los resultados indican un vacío en la atención de los enfermeros de AE y APS sobre la CPC, que puede estar relacionada con el hecho de que se trata de una enfermedad nueva o con la complejidad de su identificación, además de la falta de capacitación ofrecida por los servicios y la ausencia de una implementación efectiva de las directrices dirigidas al cuidado de la enfermedad.

En este contexto, la identificación de la CPC en los servicios de salud se ve dificultada por su carácter sistémico y la variabilidad de los síntomas, que a menudo difieren de los observados en la fase aguda de la infección por SARS-CoV-2^(11, 12)^. Los síntomas más comunes de la CPC son fatiga, intolerancia al esfuerzo, disnea y mialgia, mientras que las manifestaciones menos frecuentes incluyen diarrea, dolor abdominal, anorexia, alteraciones del olfato y del gusto, dolor de garganta, aumento o pérdida de peso, tos, palpitaciones y malestar torácico^(13)^.

La persistencia de estos síntomas puede estar relacionada con los síntomas respiratorios agudos y la hospitalización prolongada durante la fase aguda de la COVID-19. Las formas graves de COVID-19, que a menudo provocan hospitalizaciones prolongadas, pueden provocar respuestas inmunológicas intensas a la persistencia de signos y síntomas^(13)^. Además, la similitud con otras afecciones posinfecciosas, como la encefalomielitis miálgica/síndrome de fatiga crónica y la fibromialgia, influye en la definición del diagnóstico de CPC^(14)^.

Es importante destacar que la identificación de los síntomas de la CPC puede variar significativamente entre los diferentes niveles de atención médica. Los profesionales de la APS mantienen un contacto continuo y directo con los pacientes en su territorio de cobertura, ya sea a través de consultas regulares o visitas domiciliarias. Esta proximidad con el usuario favorece la identificación temprana y recurrente de los síntomas más comunes, como la tos persistente, que los pacientes suelen referir y que los profesionales de enfermería observan fácilmente, especialmente en contextos de vigilancia posinfecciosa^(15, 16)^.

Sin embargo, los déficits neurocognitivos, como la dificultad para concentrarse, la confusión mental y la pérdida de memoria, pueden interpretarse como manifestaciones más graves debido a su impacto directo en la funcionalidad y la calidad de vida, ya que interfieren en la vida cotidiana. A esto se suma una creciente difusión en la literatura sobre las secuelas neurológicas asociadas a la infección por COVID-19, lo que puede contribuir a ampliar la comprensión de la gravedad de los síntomas^(15, 16)^.

Por otro lado, la AE se centra más en atenciones puntuales, dirigidas predominantemente a casos de mayor complejidad clínica, con enfoque en afecciones específicas que requieren intervenciones diagnósticas y terapéuticas más específicas. En este contexto, los síntomas inespecíficos tienden a ser subestimados o no priorizados durante la consulta, en comparación con manifestaciones más evidentes o incapacitantes^(17)^. De este modo, las diferencias en el enfoque clínico y el vínculo de los enfermeros entre la APS y la AE influyen en la identificación de la gravedad de los síntomas de la CPC, favoreciendo la APS la observación de alteraciones sutiles y la AE centrándose en casos más complejos^(18, 19)^.

En el contexto de la APS, especialmente, el enfermero desempeña un papel multidimensional al prestar asistencia y gestionar la atención en contacto directo con los usuarios. Sin embargo, estos profesionales se enfrentan a retos significativos en la implementación de directrices, el acceso facilitado a los serviciosde salud y la realización de una acogida dirigida^(20)^. Además, cuando se enfrentan a casos clínicos de CPC que no pueden resolverse en la APS y deben ser derivados a niveles especializados de atención sanitaria, también se enfrentan a dificultades asociadas a la incertidumbre clínica, lo que tiene como consecuencia una prestación de cuidados fragmentada y mal coordinada y la falta de enfoques multidisciplinarios, descuidando las necesidades psicosociales de los usuarios y centrándose en el modelo biomédico^(21)^.

En este contexto, la falta de integración entre los niveles de atención, junto con la ausencia de flujos claros de referencia y contrarreferencia, contribuye al retraso en los diagnósticos y a la sobrecarga de la AE. En este sentido, se propone la adopción de estrategias como el uso de historias clínicas electrónicas, la estandarización de criterios clínicos para la estratificación del riesgo y la creación de consultorios especializados para facilitar la identificación temprana y la derivación adecuada de los casos. Además, la devolución de información clínica a la APS mediante la contrarreferencia es igualmente importante para garantizar la continuidad y la resolución de la atención^(22)^.

En el contexto brasileño, existe una notable dificultad en la regulación entre la APS y la AE, con una larga lista de espera para consultas y exámenes debido a la limitada resolución de las demandas en la APS. Además, la subnotificación de síntomas en los expedientes médicos ha sido un problema recurrente, que puede deberse a la falta de comunicación adecuada por parte de los usuarios o a la descripción inadecuada por parte de los profesionales de la salud. Por lo tanto, se destaca la necesidad de enfoques integrados y multidisciplinarios en la APS, especialmente para tratar las complejas CPC^(16)^.

En un estudio realizado en Bélgica, los profesionales de la APS señalan que el acceso facilitado a materiales de la literatura científica o a protocolos de manejo de la CPC es insuficiente y limitado, siendo la duración de los signos y síntomas el criterio diagnóstico más común para la identificación de los casos. Además, el conocimiento insuficiente sobre la atención y la identificación de casos de CPC en el servicio está relacionado con la falta de capacitación o información por parte de la institución de trabajo o de enseñanza sobre la enfermedad^(23)^.

En Brasil, cabe destacar la existencia del Manual para la evaluación y el manejo de las condiciones post-covid en la Atención Primaria de Salud, elaborado por el Ministerio de Salud; sin embargo, se observa una falta de difusión de este material en los servicios, dadas las dificultades de los profesionales de enfermería para identificar las manifestaciones clínicas comunes de la enfermedad, el manejo y los criterios de derivación al servicio especializado^(8)^. En este contexto, la publicación de un material no garantiza que llegue a los profesionales ni que estos lo utilicen de manera adecuada, ya que la ausencia de programas de estrategias de educación permanente dificulta el acceso e influye en la aplicación adecuada de estas directrices^(20)^.

Dicho esto, cabe destacar que el desconocimiento de las manifestaciones clínicas y la complejidad de la CPC entre los profesionales de enfermería compromete la capacidad de detección precoz, el seguimiento adecuado y el manejo clínico apropiado de los pacientes afectados. Dada la naturaleza multifactorial y a menudo inespecífica de los síntomas, la falta de conocimientos específicos puede llevar a subestimar las quejas, retrasos en el diagnóstico y fallos en la gestión de casos que requieren cuidados continuos. Esta laguna repercute negativamente en la calidad de la atención, especialmente en la APS, donde la enfermería actúa como punto de contacto inicial y elemento central en la coordinación de la atención^(17,18,19)^.

Por lo tanto, se enfatiza la necesidad de invertir en la formación continua de enfermería y en la incorporación del manual del Ministerio de Salud sobre las CPC en la rutina asistencial de la APS, que puede ponerse en práctica mediante el uso de listas de verificación clínicas estandarizadas. De esta manera, el uso sistemático de dicho manual, junto con la integración entre la APS y la AE, constituye una estrategia estructurante para calificar la atención a las personas con CPC en el sistema de salud brasileño^(22)^.

En consecuencia, el reto de la atención de enfermería no se limita a la relación entre el enfermero y el usuario, sino que también implica el cuidado y la adaptación de los propios profesionales. Además, la colaboración interprofesional y la participación activa de los usuarios en su atención también son esenciales, pero existe una considerable falta de especialistas en la área, además de la limitación de las actividades de educación permanente planificadas por los gestores, especialmente a nivel municipal^(20, 24)^.

En general, la actuación del equipo de enfermería en todos los niveles de atención se considera fundamental para ofrecer una atención integral a los usuarios con CPC, incluyendo acciones para la identificación y el seguimiento de los usuarios, orientación y apoyo, realización de actividades de educación en salud, participación en investigaciones clínicas y coordinación de la atención de forma multiprofesional, junto con médicos generales, médicos especialistas y fisioterapeutas^(24)^.

En el contexto de la APS, las necesidades de salud identificadas por el equipo de enfermería deben atenderse de manera planificada, incluyendo consultas a domicilio. Además, los profesionales deben apoyar a los cuidadores en el manejo de la CPC y otras comorbilidades adyacentes. Por otra parte, estimular la creación de grupos de educación en salud para usuarios con CPC contribuye al autocuidado y al conocimiento de los síntomas prolongados de la COVID-19^(25)^.

Por otro lado, la AE, que abarca servicios de tecnologías de complejidad media, debe contar con un equipo multidisciplinario (psicólogo, enfermero, fisioterapeuta, nutricionista y terapeuta ocupacional) para facilitar la realización de exámenes, pruebas diagnósticas y estrategias terapéuticas para el manejo de los síntomas de la CPC. Así, las competencias de la AE incluyen acoger al usuario, comprender sus necesidades y realizar evaluaciones multiprofesionales de forma integral, idealmente a través de un Proyecto Terapéutico Singular (PTS) para identificar cuidados individualizados. Además, la atención a las personas afectadas por la CPC debe compartirse con la APS, teniendo en cuenta la evolución clínica y las necesidades de los usuarios, y los casos graves deben derivarse a los servicios de urgencias^(26, 27)^.

De este modo, la derivación debe realizarse teniendo en cuenta factores biopsicosociales, a fin de contemplar la multidisciplinariedad, siendo necesario que el profesional de enfermería, además de reconocer cualquier derivación, desarrolle un plan de rehabilitación y gestión de la salud personalizado para cada individuo, teniendo en cuenta a la familia y la comunidad^(25, 28)^.

En este sentido, es necesario elaborar protocolos de atención para los servicios de salud (especialmente de la AE, ya que hay escasa producción científica sobre el manejo de la condición post-covid en este nivel de atención de salud), con el fin de facilitar la comunicación entre los profesionales y los niveles de atención, además de garantizar la calidad en la toma de decisiones, prevenir riesgos y promover innovaciones en la atención de enfermería^(29)^.

Este estudio pone de manifiesto la necesidad de desarrollar estrategias para la implementación de protocolos ya existentes y nuevos, que tengan como objetivo organizar el manejo clínico y los criterios de derivación de la CPC, dada la escasez de literatura al respecto. Además, se destaca la necesidad de que el equipo de enfermería se reconozca como coordinador de la atención a las personas afectadas por la CPC y creador de planes de atención individualizados, con énfasis en el manejo biopsicosocial del usuario, así como la urgente necesidad de fortalecer las políticas de educación permanente, especialmente a nivel municipal.

Por último, se considera una limitación de este estudio la baja participación de los profesionales de enfermería en la investigación, a pesar de que se llevó a cabo a nivel nacional y se difundió ampliamente, especialmente por el colegio profesional. Además, el estudio exploró los conocimientos de los enfermeros a través de los autoinformes de estos profesionales, lo que puede no reflejar con precisión los conocimientos técnicos reales, ya que está sujeto a sesgos de memoria, deseabilidad social o sobreestimación de los conocimientos y la práctica clínica de los encuestados. Además, el uso de un muestreo por conveniencia puede haber restringido la representatividad de los participantes, limitando la generalización de los resultados. A pesar de ello, el estudio presenta resultados sólidos, que contribuyen de manera significativa a la comprensión de los retos a los que se enfrentan los profesionales de enfermería en la identificación y el manejo de la CPC, lo que refuerza la necesidad de estrategias estructuradas de capacitación y organización de la atención.

## CONCLUSIÓN

Los resultados de este estudio indican diferencias puntuales en lo referido por los enfermeros de APS y AE, especialmente en cuanto a la identificación de manifestaciones clínicas específicas y la necesidad de derivación en casos de síntomas persistentes. Sin embargo, no se observaron diferencias significativas en las respuestas obtenidas entre los profesionales de los diferentes niveles de atención en relación con el diagnóstico, los exámenes complementarios o las acciones asistenciales.

Además, al analizar los resultados, se observa un conocimiento “básico” sobre la enfermedad (es decir, conceptos y manifestaciones comunes y de fácil designación) y su manejo, diagnóstico e identificación, ya que las respuestas contemplan la atención y el reconocimiento de la COVID-19, pero no necesariamente de la CPC. Además, se observa una falta de conocimiento sobre la CPC, ya que muchos profesionales de enfermería tienen dificultades para reconocer los síntomas, que pueden confundirse con otras afecciones.

De este modo, la identificación y el manejo clínico de la CPC se ven perjudicados por la ausencia de protocolos ampliamente difundidos, la ausencia e insuficiencia de capacitación de los profesionales de enfermería y la frágil integración entre los niveles de atención de salud. Estos factores comprometen la calidad de la atención prestada, tanto en la APS como en la AE, favoreciendo enfoques fragmentados y dificultando la respuesta efectiva a las necesidades de los usuarios.

Los resultados de este estudio destacan el papel central de la enfermería en la coordinación de la atención a las personas con CPC, poniendo de manifiesto la urgencia de desarrollar estrategias de educación permanente y capacitación continua para el equipo de enfermería, con un enfoque específico en la CPC. Esto puede garantizar la implementación efectiva de los flujos de derivación, promoviendo una atención más integrada y eficaz.

Además, las acciones integradas y multidisciplinarias, así como el fortalecimiento de la APS como puerta de entrada a los servicios de salud, son vías fundamentales para mejorar la excelencia de la atención y satisfacer las necesidades de los usuarios. Por lo tanto, este estudio contribuye a futuras investigaciones y al desarrollo de políticas públicas que busquen garantizar una atención de calidad, integrada y accesible para las personas afectadas por la CPC en Brasil.

## DISPONIBILIDAD DE DATOS

Todos los datos que respaldan los resultados de este estudio están disponibles en este artículo.
